# Current Applications and Discoveries Related to the Membrane Components of Circulating Tumor Cells and Extracellular Vesicles

**DOI:** 10.3390/cells10092221

**Published:** 2021-08-27

**Authors:** Luis Enrique Cortés-Hernández, Zahra Eslami-S, Bruno Costa-Silva, Catherine Alix-Panabières

**Affiliations:** 1 Laboratory of Rare Human Circulating Cells (LCCRH), University Medical Centre of Montpellier, CEDEX 5, 34295 Montpellier, France; le-cortes-hernandez@chu-montpellier.fr (L.E.C.-H.); z-eslami-samarin@chu-montpellier.fr (Z.E.-S.); 2 CREEC/CANECEV, MIVEGEC (CREES), Université de Montpellier, CNRS, IRD, 34000 Montpellier, France; 3 Champalimaud Research, Champalimaud Centre for the Unknown, 1400-038 Lisbon, Portugal; bruno.costa-silva@research.fchampalimaud.org

**Keywords:** liquid biopsy, extracellular vesicles, circulating tumor cells

## Abstract

In cancer, many analytes can be investigated through liquid biopsy. They play fundamental roles in the biological mechanisms underpinning the metastatic cascade and provide clinical information that can be monitored in real time during the natural course of cancer. Some of these analytes (circulating tumor cells and extracellular vesicles) share a key feature: the presence of a phospholipid membrane that includes proteins, lipids and possibly nucleic acids. Most cell-to-cell and cell-to-matrix interactions are modulated by the cell membrane composition. To understand cancer progression, it is essential to describe how proteins, lipids and nucleic acids in the membrane influence these interactions in cancer cells. Therefore, assessing such interactions and the phospholipid membrane composition in different liquid biopsy analytes might be important for future diagnostic and therapeutic strategies. In this review, we briefly describe some of the most important surface components of circulating tumor cells and extracellular vesicles as well as their interactions, putting an emphasis on how they are involved in the different steps of the metastatic cascade and how they can be exploited by the different liquid biopsy technologies.

## 1. Introduction

Cancer starts as a local uncontrolled expansion of cells that is caused by genetic and epigenetic alterations thriving in specific microenvironments (e.g., chronic inflammation) [1,2], but other factors contribute to make cancer a lethal disease. Indeed, cancer progressively becomes a systemic disease that spreads through the body in a process called the metastatic cascade [3]. Metastasis formation in distant organs and systemic effects are the main causes of death in most patients with cancer [4].

Despite the lack of a clear holistic view on the metastatic cascade and the systemic effects of cancer, several cancer analytes and biomarkers have been described [5]. These analytes provide valuable information on disease status and progression [6,7,8,9]. Secreted proteins present in blood, for instance prostate specific antigen and carcinoembryonic antigen, are used in clinical laboratory tests [10]. These biomarkers are related to the tumor size and progression (because they are secreted by specific cancer cells) and bring useful information for the clinicians [11,12,13]. However, they are not causally related to the metastatic cascade and the cancer systemic effects. Therefore, much research effort has been focused on the identification of more useful markers in blood that provide direct/causal knowledge on the cancer pathobiological processes [3]. This has led to the emergence of the liquid biopsy field, in which different complementary circulating biomarkers from body fluids, mainly blood, are investigated using minimally invasive methods [5,14] (Box 1).

Box 1 **Definitions in liquid biopsy.**

**Liquid biopsy:** In cancer: the term “liquid biopsy” was coined for the first time in 2010 [15] and describes the minimally invasive analysis of circulating biomarkers derived from primary and/or metastatic tumors. These biomarkers give real-time complementary information on the tumor and can be found in any physiological and pathological body fluids (e.g., blood, ascites, urine, sputum, bone marrow) [5,16].

**Circulating tumor cells (CTCs):** CTCs are more aggressive cancer cells that have been released actively by the primary and/or metastatic tumor(s). These cells can be found in physiological and pathological fluids but are mainly detected in the blood circulation. Different approaches for their enrichment and isolation have been developed, based on CTC physical and biological properties, to distinguish them from the surrounding normal cells. Currently, the CellSearch® system is the only method cleared by the U.S. Food and Drug Administration for CTC enumeration in metastatic breast, prostate and colorectal cancer. This method includes (i) a positive enrichment step based on EpCAM expression at the CTC surface, and (ii) a detection step based on the expression of a panel of cytokeratins (CK9, 18 and 19), DAPI staining (nucleus) and lack of CD45 expression (specific leukocyte marker).

**Extracellular vesicles (EVs):** EV is an umbrella term for all kinds of vesicles released by cells (e.g. microvesicles, exosomes and oncosomes). This term has been increasingly used due to the current limitations to specifically isolate only one vesicle type with high purity, and as a way to recognize the high heterogeneity of the material obtained with current isolation methods [17,18]. It is acknowledged that different EVs have specific origins and biological functions. For instance, exosomes are small EVs (between 35 and 150 µm in diameter) of endosomal origin, with roles in the pre-metastatic niche establishment and cancer cell organotropism [19].

Circulating cell-free nucleic acids (cfNA), released by cancer cells, are an example of liquid biopsy analytes. Analysis of cfNA allows for the detection of driver mutations or genetic alterations that might guide therapy decision-making or contribute to the early diagnosis of cancer [20]. However, the mechanisms underlying cfNA origin in blood are not clear. It has been suggested that cfNA are actively released by cells [21]; however, most studies have shown that circulating cell-free DNA (cfDNA), the most studied cfNA, originates from apoptotic cells because the cfDNA fraction size correlates with what is expected from apoptotic degradation [22]. Therefore, the release of circulating tumor DNA (ctDNA), which correspond to the cfDNA fraction released by cancer cells, might just be a consequence of cancer progression (or caused by therapy [6,23,24]), without any specific role in the disease course [5]. 

Conversely, other liquid biopsy analytes might not only reflect cancer progression but also play a key role in the biological mechanisms of the systemic disease [19,25,26,27,28]. Two clear examples of such analytes are circulating tumor cells (CTCs) and extracellular vesicles (EVs) (Box 1). Even if one is a cell and the other one a vesicle released by a cell, these analytes share many similarities, and the most important one is that they both have a phospholipid membrane (Box 2), which differentiates them from classical tumor biomarkers (e.g., prostate specific antigen, carcinoembryonic antigen) and cfNA. Specific patterns of proteins, phospholipids and even nucleic acids on the membrane surface of these analytes, which can be similar or different to the ones on the tumor cell surface, may hold the key to understanding the systemic dissemination of cancer.

Box 2 **Cell membrane.**

The cell (or plasma) membrane preserves the cell structure and is an important barrier between the internal and the external environment. Due to the plasma membrane selective permeability, only some substances (e.g., hormones, ions, enzymes, growth factors and other molecules) can enter and exit the cell. Cells can also transfer endogenous signals and molecules to other cells and to the micro- and macro-environment. Indeed, cell membranes play crucial roles in cellular signaling and communication. The plasma membrane is also strongly involved in the metastatic process. The establishment of metastases requires complex interactions between stroma, tumor cells, normal cells and the extracellular matrix that are mediated mainly by the cell membrane [29]. Unraveling specific expression patterns and interactions of cell membrane proteins, phospholipids and even nucleic acids may help to better understand the metastatic cascade. Phospholipids are a major component of the cell membrane. They are lipid molecules made of phosphate groups (hydrophilic) and two tails of fatty acids (hydrophobic) that allow formation of a bilayer with an outer leaflet in contact with the extracellular matrix and an inner leaflet in contact with the cytoplasm. The cell membrane also contains glycolipids and sterols. One of the most important sterols is cholesterol, which regulates the cell membrane fluidity in animal cells and contributes to maintain its permeability. Lastly, proteins present in the plasma membrane have a wide range of functions, such as receptors that bind to signal molecules, ion channels and structural proteins that can attach to the extracellular matrix or that mediate cell–cell interactions [30,31].

The roles and clinical applications of each liquid biopsy analyte have been fully reviewed elsewhere as well as the characteristics of cargoes and their contents [16,20,32,33,34,35,36,37,38]. In this review, we focused on the cell membranes of CTCs and EVs, and specifically on how interactions of cell surface proteins, phospholipids and nucleic acids could play a key role in the different steps of the metastatic cascade.

## 2. Cell Surface Proteins

Cell surface proteins guide most molecular and cellular interactions (i.e., communication and adhesion) [39,40,41]. Moreover, they have functions related to cell integrity, for example, by working as pumps or channels for the transport of many molecules and substances [42]. The histopathological characterization of different cancers types is often based upon or supported by demonstrating the expression of specific proteins at the cellular membrane [43,44]. Proteins expressed at the tumor cell surface can also be detected in CTCs or EVs where they can mediate interactions with other blood components, such as platelets, that contribute to the metastatic cascade [45]. Moreover, the presence of specific surface proteins can be exploited for the enrichment, capture, identification and characterization of these analytes [18,46,47]. Lastly, many targeted therapies rely on the detection of specific proteins in cancer tissue biopsy samples, and the same strategy could be transferred to liquid biopsy [48]. In the next paragraphs, we will describe some of these cell surface proteins.

Epithelial cell adhesion molecule (EpCAM) is overexpressed in many carcinoma types with stem cell features [49]. EpCAM is currently used for CTC detection and enrichment by the CellSearch^®^ system, the only method cleared by the Food and Drug Administration (FDA) in the U.S. for the analysis of CTCs as a prognostic tool in metastatic breast, prostate and colorectal cancer [46,50,51,52]. Moreover, the recent French interventional clinical trial “STIC CTC” showed that in women with breast cancer, the detection of ≥5 EpCAM-positive CTCs can guide towards a more aggressive treatment (chemotherapy instead of endocrine therapy) [9]. EpCAM has also been used to detect EVs, and their enumeration is correlated with prognosis [53]. 

Nevertheless, in the circulation, CTCs undergo phenotypic changes (for instance, loss of epithelial marker expression) to survive the fluid shear dynamics of blood and lymph [54]. The expression of adhesion proteins on CTCs can be modified also during the epithelial–mesenchymal transition (EMT) when proteins implicated in epithelial cell polarity and integrity are frequently downregulated [55]. One key change during EMT is the “cadherin switch” from E-cadherin to N-cadherin, which is associated with a mesenchymal phenotype [56,57]. Moreover, metastasis-initiator CTCs are characterized by high phenotypic plasticity, leading to the re-expression of some epithelial markers that facilitate metastatic tumor initiation in a distant organ [58,59]. In the bloodstream, platelets interact with CTCs and release transforming growth factor-β that promotes EMT [60]. These features ultimately result in higher CTC proliferation and survival [61,62,63]. It has been suggested that not all cancer types similarly rely on EMT [54], as exemplified by the different mean number of EpCAM-positive CTCs detected in cancer types that originate from the same organ but with different biological features (e.g., high CTC number in patients with small cell lung cancer and low CTC number in patients with non-small-cell lung cancer) [64,65].

During EMT, not only the expression of cadherin proteins and EpCAM is modified, but also the expression of other cell interaction proteins, such as integrins [41]. The family of integrins includes 24 different transmembrane heterodimers with different functions. Integrins are the main cell adhesion receptors at the cell surface [40]. In non-cancerous cells, the double function of these proteins, for adhesion and signaling, limits cell migration, and loss of cell anchorage, mediated by interaction of integrins with the extracellular matrix, induces anoikis, a specific apoptosis type [66]. In CTCs, EMT might increase the expression of integrins related to cell migration and survival. For example, overexpression of integrin β1 is associated with resistance to anoikis, which might be explained by integrin signaling that activates proliferative pathways [67]. 

Furthermore, some of the CTCs that survive the circulation stress (e.g., blood pressure, immune cell attacks) might extravasate at specific body locations (organs or tissues). To achieve this step, CTCs must interact with other cell types through specific proteins. In vivo studies using zebrafish cancer models showed that the glycoprotein CD44 and the integrins αvβ3 and α5β1 are required for weak (CD44 and αvβ3) and strong (α5β1) interactions between CTCs and endothelial cells [68]. In mice models, integrin αvβ3 inhibition with specific antibodies correlates with tumor regression [69]. In addition, neuropilin-2 (NRP2) on CTCs interacts with integrins on the endothelial cell surface to promote cell attachment [70]. Integrins are involved not only in CTC dissemination. Indeed, in small EVs, differential expression of integrins dictates the metastasis organotropism: integrins α6β4 and α6β1 are associated with lung metastases, and integrin αvβ5 with liver metastases [19]. An in vitro study in breast cancer cells showed that integrin β3 expression at the EV surface promotes their uptake, by interacting with heparan sulfate proteoglycans [71,72]. Moreover, in murine models of non-alcoholic steatohepatitis (NASH), integrin β1-enriched EVs are released by hepatocytes upon lipotoxic stress. These EVs mediate the recruitment of monocyte-derived macrophages, which is the first step in NASH development. Integrin β1 blockade by antibodies inhibits NASH progression in these models [73]. Moreover, EVs secreted by lymphocytes can reflect the inflammation status in chronic hepatitis C, and represent a candidate biomarker of NASH [74]. Furthermore, the expression of integrins, as well as other proteins, in EVs have shown the potential to identify the primary site of cancer in plasma samples [75].

The expression of different integrins in platelets also has a role in cancer progression; for example, α6β1 and αIIbβ3, might participate in the platelet–tumor cell interaction and in cancer metastasis formation [76]. Targeting these receptors efficiently reduces experimental metastasis formation. The cancer cell capacity to adhere to the endothelium is promoted by mechanisms that could be linked to integrin αIIbβ3 receptor expression on platelets [76]. Briefly, interaction of this receptor with integrin αvβ3 on CTCs leads to platelet activation and increased coagulation [68] that mediates stable CTC–endothelium bonds and facilitates their extravasation. Additionally, in mice with platelets that lack αIIbβ3, metastatic foci are reduced in the bone [77]. Moreover, blockage of αIIbβ3 with eptifibatide (a specific platelet inhibitor) can reduce breast cancer cell adhesion and migration [78].

Stem cell surface proteins also play a role in metastatic progression and interact with integrins and other proteins. For example, CD44 is implicated in CTC interactions with endothelial cells and is one of the most commonly expressed stem cell markers [79]. CD44 interacts with hyaluronic acid and osteopontin, which are components that can facilitate the further attachment to distant tissues; also CD44 homophilic interactions can maintain the formation of a CTC-cluster [80]. Moreover, the isoform CD44v6 correlates with colorectal cancer (CRC) and prostate cancer progression [81,82], and CD44 correlates with metastasis formation [83]. It has been shown that detection of CD44v6 on the CTC surface predicts the radiological response in metastatic CRC [84]. Additionally, in vitro studies found that more aggressive cancer cells can use EVs carrying CD44 or CD44v6 to transfer a migratory and invasive phenotype to less aggressive cancer cells [85,86] in ovarian and CRC models. EVs also help to maintain stemness during embryogenesis. Fibronectin associated with EVs enables the interaction with integrins on the surface of embryonic stem cells and then induces FAK, resulting in the limitation of cell differentiation [87]. Similar mechanisms might be used by cancer cells to maintain their high proliferation rates. Expression of CD44 on EVs has been associated with breast cancer recurrence [88]. CD44 variants also act as major platelet E-/L-/P-selectin ligands in CRC cells [89,90]. It has been shown that platelets increase the levels of CD44 and tissue factors on CTCs and also act as chemoattractants to tumor cells and induce sphere (cluster) formation [91]. In mice, intravital microscopy showed that liver colonization by platelets depends mainly on Kupffer cells (the liver macrophages) at early and late stages of NASH and involves hyaluronan–CD44 binding [92]. CD44 expression is one example of how a surface protein is shared on CTCs and EVs, in which the same protein interactions can lead to different roles in cancer progression. 

Proteins related to resistance to cancer therapy, such as molecular pumps that mediate the drug passage through the cell membrane, have been much studied [93,94,95]. For example, expression of multi-drug resistance-associated protein 1 (MDP1) or of the ABCC1 glycoprotein on the CTC surface has been associated with therapy resistance and CRC progression [96]. Similarly, therapy resistance-associated proteins have been detected on EVs, and it has been suggested that cancer cells can transfer these proteins to other cancer cells through EV-based communication [97,98,99]. Likewise, MDP1 or P-glycoprotein-positive EVs could play a role in the efflux of chemotherapeutic agents, thus decreasing their intracellular concentration [100]. Moreover, EVs might act as a decoy target for immunotherapies and induce immunotherapy resistance. For instance, in vitro studies using HER2-overexpressing breast cancer cell lines showed that HER2-positive EVs can interfere with anti-HER2 antibodies, reducing their effects [100,101].

Other target therapy markers are expressed at the surface of CTCs and EVs. For instance, programed death-ligand (PD-L1) can be detected on the CTC surface in many cancer types. Our group was the first to show the feasibility of PD-L1 detection of breast cancer CTCs using the standard CellSearch^®^ system [102], and we confirmed these first data in breast cancer [48] and in non-small-cell lung carcinoma [103]. PD-L1 expressed at the platelet surface might contribute to cancer cell immune invasion, thus explaining why some patients with PD-L1-negative cancer still respond to PD-L1 inhibitors [104]. PD-L1 has been detected also on EVs [105] where it plays a key role in immunotherapy resistance [106].

**Figure 1:**
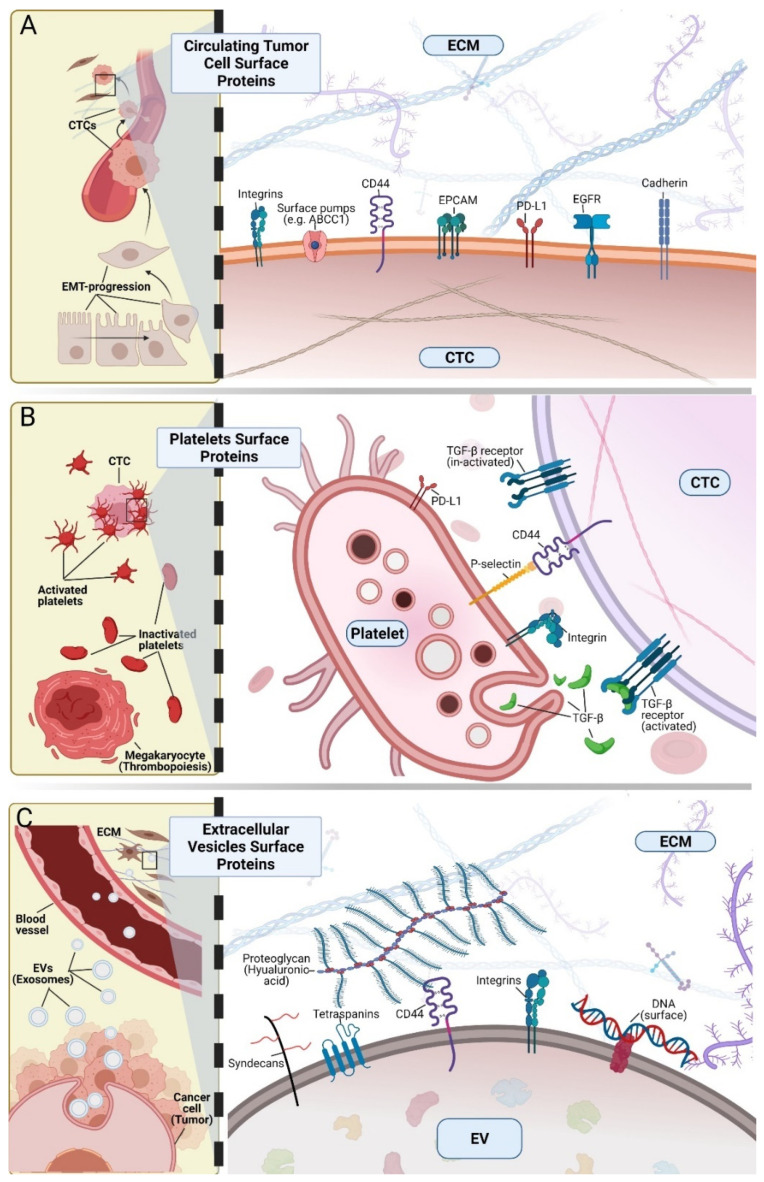
Surface proteins and their interactions in circulating tumor cells (CTCs), platelets and extracellular vesicles (EVs). (**A**) Surface proteins on CTCs. During cancer progression, cancer cells can go through a reconfiguration of their protein expression profile. For instance, in carcinomas, during epithelial-to-mesenchymal transition (EMT), loss of epithelial features (e.g., downregulation or loss of E-cadherin) allows the dissemination of tumor cells, as well as interactions with endothelial cells and the extracellular matrix (ECM). Relevant examples are interactions with CD44, integrins and EpCAM. Moreover, proteins already expressed in the primary tumor are also detected at the CTC surface (e.g., PD-L1, EGFR). (**B**) Platelet surface proteins are related to the interaction with CTCs in the bloodstream. Platelets are derived from megakaryocytes in the bone marrow and might be primed by cancer cells to facilitate interactions with CTCs. For instance, P-selectin expressed at the platelet surface can interact with CD44 on the CTC surface. Moreover, platelets release transforming growth factor β (TGF-β) that promotes EMT. Platelets can also express PD-L1 that might act as an inhibitor of the immune response against CTCs. (**C**) The EV surface protein profile is related to their endosomal origin (e.g., tetraspanins or syndecans); however, EVs also carry at their surface proteins that facilitate their uptake or interaction with the ECM. For example, (i) CD44 interacts with hyaluronic acid in the interstitial ECM of distant organs, (ii) integrins define EV organotropism, contributing to the establishment of the pre-metastatic site. Moreover, EV proteins are associated with other components such as DNA.

In CTCs, targetable surface proteins might harbor mutations, like the tumor of origin. For instance, in-frame deletions in exon 19 and the missense mutations L858R and T790M in EGFR have been identified in non-small cell lung cancer and also in CTCs. Interestingly, in some cases, genotyping is not 100% identical between tumor biopsies and CTCs; however, it is unclear how such discrepancies might relate to response to therapy [107,108,109]. Other targetable membrane proteins, such as HER2 and estrogen receptor in breast cancer, also show similar discrepancies between tissue and liquid biopsies [32]. Targetable genetic alterations (e.g., EGFR mutations) can be detected also in EVs, highlighting the possibility to improve the current methods based on cfNA to detect these genomic aberrations [7,110,111]. However, it is unclear whether EVs harbor the modified proteins on their surface and whether this modifies their biological activity. Finally, the identification on the surface of liquid biopsy analytes of markers with proven clinical utility in tissue biopsies might help to monitor therapy resistance development and to understand the underlying mechanisms. However, more interventional clinical trials are needed to precisely determine their clinical utility for stratifying patients, for targeted therapy monitoring and for identifying resistance mechanisms. All aforementioned interactions in CTCs, platelets, and EVs through surface proteins are summarized in Figure 1.

## 3. Membrane Lipids

There are thousands of different phospholipids and lipids in the cell membrane. Cell membranes are made of a lipid bilayer in which lipids are asymmetrically distributed: sphingolipids and phosphatidylcholine (PC) are present mostly in the outer leaflet, while phosphatidylserine (PS), phosphatidylethanolamine (PE) and phosphatidylinositol (PI) are mainly in the inner leaflet of the membrane [112]. Lipids provide the membrane structure, and they also are implicated in several metabolic, signaling and cell-to-cell interaction pathways. Moreover, in some cells, during apoptosis and aging, PS is translocated from the inner to the outer leaflet, where it acts as an “eat me” signal for macrophages and can overcome the “not eat me” signal related to CD47 expression [113]. In analogy, cancer cells might (i) increase the amount of PC and sphingolipids and (ii) maintain PS in the inner leaflets to improve their survival and proliferation. These mechanisms might also be used by CTCs and are relevant for EV biogenesis. 

The few available lipidomic studies in EVs have shown an enrichment of cholesterol, PS, sphingomyelin and glycosphingolipids; however, better EV isolation methods need to be developed to improve their purification and identification [114,115]. This is particularly important because it has been suggested that the EV lipid profile could be used as a diagnostic/prognostic biomarker for different diseases [116,117,118]. Indeed, with the currently available isolation methods, it is difficult to precisely assess the lipid composition of the different EV types because of their small size and yield heterogeneity. This is due to the existence of different EV populations with some overlap in physical features, biogenesis and molecular function. In addition, recent studies have questioned the standard methods for separation and characterization of EV subpopulations. In conclusion, currently, no robust technique is available for the complete separation of the different EV subtypes [21,119,120,121]. Nevertheless, the observed enrichment in PS could be explained by (i) the high amounts of apoptotic vesicles isolated with other vesicles; and (ii) the high PS fraction at the EV surface that facilitates their uptake by other cells. As better isolation and characterization methods are developed, these issues will soon be solved.

Currently, the study of CTC phospholipid composition is highly challenging because CTCs are “rare events” in blood, and they cannot be captured in sufficient numbers for lipidomic analyses. Permanent patient-derived CTC lines can provide enough cells for phospholipid composition characterization [122,123], but no study has tried to characterize their composition yet. Indeed, most studies on the role of phospholipids in the metastatic cascade have been done in vitro. For example, a study found that PE and PI levels are significantly lower in a breast cancer cell line with high than in the one with low metastatic potential [124].

**Figure 2:**
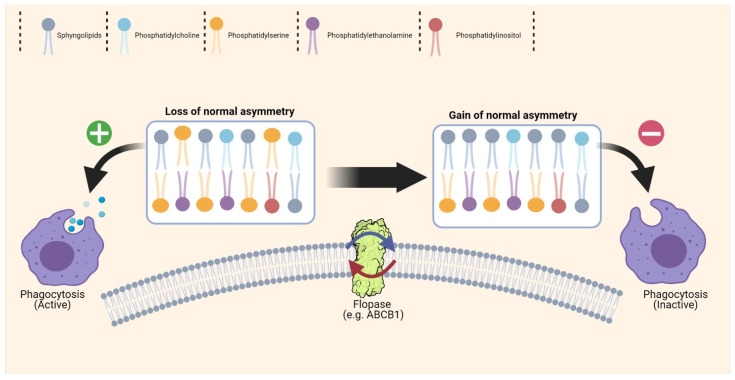
Cell membrane phospholipid composition. In physiological conditions, the cell membrane is characterized by an asymmetric distribution of phospholipids, but this feature is lost during normal cell ageing. For example, in erythrocytes and platelets, phosphatidylserine relocation from the inner leaflet to the outer leaflet of the membrane facilitates the phagocytosis of apoptotic cells and older cells. Normally, the membrane lipid distribution profile is regulated by specific proteins, such as flipases and flopases, and cancer cells might overexpress these proteins to support their high proliferation rate and to avoid phagocytosis. This mechanism might also be necessary for the biogenesis of extracellular vesicles to avoid their unspecific uptake. Nevertheless, in cancer cells, loss of the lipid distribution asymmetry might help to interact with other cells or might facilitate platelet activation on their surface.

Flipases, flopases and scramblases are the proteins in charge of regulating the cell membrane asymmetric phospholipid composition. Their implication in EV biogenesis is not clear. An in vitro study showed that knockdown of the flipase ATP9A reduces the total number of EVs in a hepatoma cell line [125]. These proteins might have an important role in exosome survival in the bloodstream. Indeed, these vesicles are formed by in-budding of the endosome membrane [37], and the maintenance of the asymmetric phospholipid composition is fundamental to keeping surface proteins in the right orientation and to limit the unspecific uptake of these vesicles by macrophages (Figure 2).

In cancer cells, PS percentage in the outer leaflet of the membrane is increased, despite the mechanism to keep the normal asymmetry [126,127,128]. The altered distribution of membrane phospholipids in cancer cells promotes the coagulation cascade activation. Once on the outer membrane leaflet, anionic PS (i) create a negatively charged surface that promotes binding to factors Xa and Va (key elements in the coagulation cascade), initiating the assembly of the prothrombinase complex, and (ii) support the prothrombin conformational change (conversion from prothrombin to the partially active intermediate meizo-thrombin) that results in the activation of their proteolytic activity. This leads to thrombin deposition and clot formation [129]. Therefore, understanding the phospholipid asymmetry or redistribution might help to understand platelet function, especially in cancer. Moreover, platelets can incorporate exogenous phospholipids into their membranes [130]. PS enrichment generally suppresses platelet function, and although PE enrichment does not affect platelet α- and δ-granule secretion, it activates platelet thrombotic function [130]. 

## 4. Surface Nucleic Acids

**Figure 3:**
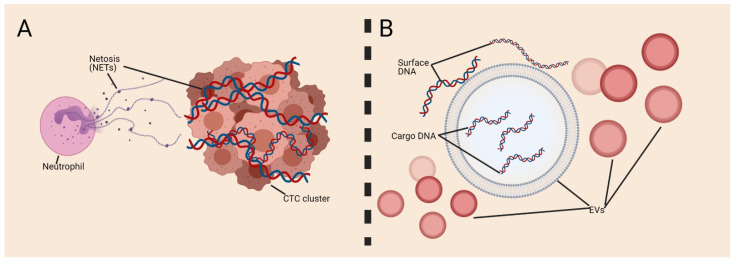
Surface DNA. Little is known about DNA interaction with circulating tumor cells (CTCs) or extracellular vesicles (EVs). (**A**) Neutrophils travel with CTC clusters in the bloodstream; this interaction might be facilitated by netosis with the formation of neutrophil extracellular traps (NETs) of DNA expelled from the neutrophil nucleus. (**B**) DNA may be present on the EV surface; however, few studies have been performed to understand this phenomenon.

It has been suggested that not all nucleic acids in blood or fluids can be found as a cfNA, and some studies have described the presence of cell membrane-associated DNA and RNA [104,105,106]. No study has documented the presence of DNA on the CTC surface yet; however, an in vivo study in breast cancer demonstrated that CTC clusters can be formed in association with neutrophils and that these clusters increase CTC metastatic potential [131]. It has been shown in vitro that neutrophils trap CTCs using neutrophil extracellular traps (NETs) [132,133] in which DNA is released as a net to capture CTCs [134]. Noteworthily, platelets also induce NET formation [135], and this might promote the establishment of a pro-thrombotic state [136], leading to CTC cluster formation or endothelial injury. These studies suggest that NETs can facilitate the survival of cancer cells in a dormant state and even promote metastasis formation. Some studies reported that DNA might be present on the EV surface [137]. For instance, an in vitro study using the Jurkat cell line found higher amounts of DNA at the EV surface after exposure to antibiotics (ciprofloxacin) with affinity to fibronectin [137]. CTCs might reconfigure not only their external morphology but also their internal composition. For instance, it has been suggested that DNA might be actively released, increasing the cell flexibility. EV constitution might be explained by the fact that cells quickly diminish their surface and internal components [138]. However, the role of DNA on the EV surface has not been elucidated, and this observation has not yet been confirmed in vivo (Figure 3).

Similarly, it is not known whether RNA is present on the surface of CTCs or EVs, but a recent study reported the presence of RNA associated with membranes in vitro [139], and another report demonstrated the existence of glycosylated RNA at the cell surface that interacts with members of the Siglec receptor family [140]. The implication of this finding in cancer progression remains unclear. 

## 5. Complexity and Complementarity of EVs and CTCs

EVs and CTCs share many membrane surface features, but the exact composition of their membrane is dictated by their biogenesis mechanism [37,141]. Consequently, differences can be observed between CTCs and EVs, and also within different subpopulations of the same analyte. Indeed, the expression of different proteins on EVs of different sizes, and the expression of tetraspanins on EV surfaces is not exclusive to small EVs [18,119]. Moreover, within the small EVs that express tetraspanins, there are subpopulations with different expression patterns [18]. Similarly, the existence of two subpopulations of small EVs (Exo-S and Exo-L) has been demonstrated, in which Exo-S display specific features related to their endosomal origin, whereas Exo-L are enriched in proteins linked to the cell membrane and intracellular compartments of the Golgi apparatus [142]. In CTCs, variations in protein expression are related to their clonal evolution and EMT. Furthermore, in CTC clusters, epithelial proteins are strongly expressed to promote cohesion within the cluster [25], for instance cell-to-cell adhesion proteins (e.g., claudins, plakoglobins and keratins) [143,144,145]. Moreover, intercellular junctions formed by VCAM-1 on CTCs facilitate interactions with neutrophils in heterotypic clusters [131]. These studies highlight that the different surface protein expression profiles in EVs and CTCs bring complementary information on the origin and function of these analytes. 

## 6. Conclusions

Most of the current therapeutic strategies target primary and/or metastatic cancer cells. However, many metastatic process steps are still ignored. Surface components of liquid biopsy analytes might represent future targetable markers to block the metastatic process, and more importantly the systemic effects of cancer, which is the ultimate cause of death in most patients with cancer. Future studies should clearly identify the function of surface components and associate them with clinical outcomes. Moreover, the identification of the roles of these analytes in cancer progression could open new opportunities for the development of novel therapies. As methods for the analysis of liquid biopsy analytes are improved, therapies that target them will have the advantage of a coherent direct real time follow-up.
